# Train Climbing—A new old trend in adolescents: Treatment of high voltage injuries and planning of a pilot project to raise awareness

**DOI:** 10.1007/s00508-024-02399-1

**Published:** 2024-08-22

**Authors:** Viktoria Koenig, Philipp Tratnig-Frankl, Anna Pittermann, Marita Windpassinger, Julian Joestl, Oskar Aszmann

**Affiliations:** 1 Division of Plastic, Aesthetic and Reconstructive Surgery, Medical University of Vienna, Waehringer Guertel 18–20, 1090 Vienna, Austria; 2 Division of General Anaesthesia and Intensive Care Medicine, Medical University of Vienna, Waehringer Guertel 18–20, 1090 Vienna, Austria; 3 Private Clinic Priv.-Doz. Dr. Julian Joestl, PhD, MSc., Spitalgasse 19, 1090 Vienna, Austria

**Keywords:** Train surfing, Burn injuries, Burns, Awareness project, Burn surgery

## Abstract

**Introduction:**

An increasing number of high voltage electric burn injuries in a typically younger patient collective of train surfers and climbers at our level I center for burns was recognized. The purpose of this study was a retrospective data evaluation and as a consequence the implementation of an awareness program against train surfing.

**Material and methods:**

In a retrospective analysis of prospectively collected data, 17 patients with high voltage injuries, who had been treated at our unit between January 2022 and January 2023, were identified. Of these patients seven were treated for injuries due to train surfing or climbing and therefore included in this study. The patients were assessed clinically for total burn surface area (TBSA), degree of burn, associated Injuries, hospital length of stay, number and type of surgeries (fasciotomy, minor/major amputations, defect coverage split skin graft or flaps).

**Results:**

A total of seven males formed the basis of this report with an average age of 17.7 years (range 14–21 years). The highest ABSI (Abbreviated Burn Severity Index) score was 12, leading to the death of the 21-year-old patient who had 80% TBSA as well as multiple comorbidities including severe brain damage. The mean duration of stay at the intensive care unit (ICU) was 24.8 days and the mortality rate was 14.29%.

**Conclusion:**

This study highlighted the severity of injuries, with a mean TBSA of 41.42% and a mortality rate of 14.29% among the study population. Train climbing and surfing patients presented with severe injuries and fatal long-term consequences. A pilot project involving several stakeholders was initiated in order to raise awareness of the dangers of electric arcs and the risk involved.

## Introduction

The number of young people seeking thrills and adventure in train surfing and train climbing is rising. Both activities are considered as extremely dangerous and can lead to serious injuries mostly due to high voltage electric injuries and burn injuries. High voltage electric injuries showed a 90% male predominance over the last few decades. In train surfing-related injuries, the gender distribution was similar but the age group concerned was younger [1, 2].

In the current literature there are strategies for the treatment of burn patients, but high voltage injuries should be viewed and managed as a multisystem injury, as there is virtually no organ that is protected against it [3–6].

In comparison to normal burn patients, 3rd degree burn injuries are found, marking the entry and exit point of the current, often resulting in amputations. Furthermore, muscle damage leading to rhabdomyolysis and kidney failure is common. Patients suffer from cardiac arrest and arrythmia. Secondary trauma after falls from a train is common, leading to traumatic brain injury, fractures, spinal injuries and paraplegia [4, 7].

When examining the existing literature on surgical treatment, there are no universally accepted recommendations [7]. This might be due to the fact that the patients all have such different injury patterns. Polytrauma treatment, decompression against compartment syndrome and proper defect coverage after debridement is necessary [4]. Consequently, it is pertinent to assess outcomes by focusing on the affected extremities, considering decompression, amputation, and associated complication rates [8, 9]. While the majority of reports compare decompression rates categorized by patients, only limited data are available regarding the ratio of decompression to amputation [8–10]. Addressing soft tissue coverage following high voltage injury poses challenges, particularly when local tissue reserves are limited [9, 11, 12]. Microsurgical reconstruction emerges as a valuable tool in addressing wound-related issues [13, 14]. Among the structures vulnerable to damage after high voltage injury, the body’s vasculature is frequently affected, leading to progressive tissue necrosis and complicating reconstructive management. Determining an optimal timeframe for microsurgical reconstruction proves challenging: early coverage may be linked to incomplete debridement, while delayed reconstruction carries the risk of tissue desiccation and infection [14]. Depending on the observed complication rates, some authors advocate either approach [15].

The purpose of this study was to give an overview of high voltage injuries and compare the outcomes to the current literature.

## Material and methods

### Patient identification and data collection

Following local ethics committee approval (ethics committee approval number: 1384/2023), the medical records of a consecutive series of seven patients with high voltage electric burn injuries due to train surfing or climbing who had been prospectively entered in the burn database over a period from January 2022 and January 2023 were reviewed. Patients with electrical injuries due to work reasons or low voltage injuries (< 1000V) have been excluded.

Clinical data were reviewed to identify patient demographics (age), mechanism of injury, side of injury, average surgery time, overall lengths of hospital stay (LOS) total burn surface area (TBSA), grade and depth of burn, associated injuries, number and type of surgeries (fasciotomy, minor/major amputations, defect coverage split skin graft or flaps), and mortality rate.

### Statistics

Statistical analysis was undertaken using R Version 3.1.1 SPSS (Armonk, NY, USA).

## Results

### Demographic characteristics

A total of seven males formed the basis of this report with a mean age of 17.7 years (range 14–21 years) (Table 1).

**Table 1:** Demographic data

Patient	1	2	3	4	5	6	7
Age (years)	16	19	17	21	21	16	14
Sex	m	m	m	m	m	m	m
ABSI	6	2	6	12	4	6	4
%TBSA	35	5	50	80	35	50	35
Entry mark	Back	NA	Head	Head	Head	Head	RA
Exit mark	RL	LL	LL, RL	LL, RL	LL, RL	RL	LL
Days at ICU	23	3	45	1	20	42	40
Days at hospital	31	19	64	1	36	108	40*
Death	0	0	0	1	0	0	0

*RL* right leg; *LL* left leg,* RA* right arm, *LA* left arm, *NA* data not available, *ABSI* Abbreviated Burn Severity Index, *TBSA* total burn surface area, *ICU* intensive care unit

*Patient was an American citizen and was transferred to the USA

The injury mechanism was a high voltage injury due to a light arc while train climbing or train surfing in all 7 patients.

The highest ABSI (Abbreviated Burn Severity Index) score was 12, leading to the death of the 21-year-old patient who had 80% TBSA as well as multiple comorbidities including severe brain damage.

The mean ABSI score was 5.7 (range 2–12).

The mean TBSA was 41.42% (range 5–80%) and all of the patients suffered from 2b or 3rd degrees burn injuries.

Regarding the entry and exit marks we found 4 patients with entry marks on the head (57.14%), all patients (100%) had exit marks on the legs. One entry mark was found on the back and another on the right arm of the patient.

The mean duration of stay at the intensive care unit (ICU) was 24.8 days (range 1–45 days), mean stay at hospital was 42.71 days, (range 1–108 days, including stay at ICU), 1 patient died on the day of admission and another patient was transferred to another hospital after leaving the ICU.

1 patient died at the day of admission due to severe brain damage which leads to a mortality rate of 14%.

### Surgical procedures and complications

Fasciotomy was carried out on the other 6 patients in the upper and/or lower extremity. In total 5.28 surgeries (range of 0–12) were performed per patient.

All these 6 patients underwent defect coverage with a split skin graft (SSG), 1 local flap was performed (latissimus dorsi flap (LD) for the coverage after amputation of the right arm), and 4 free flaps were performed (2 LD for coverage of the scalp, 1 Anterolateral tigh flap (ALT) for coverage of the scalp, 1 LD for pretibial defect coverage).

One patient had a forefoot amputation as well as the amputation of 2 toes on the other foot, regarding major amputations we recorded one amputation of the right arm and 2 amputations of one lower extremity, including one Pirogoff amputation. In total the patients underwent 5.28 surgeries (range 0–12) (Table 2).

**Table 2:** Surgical procedures

Patient	1	2	3	4	5	6	7
# of surgeries	3	4	12	0	3	8	7
SSG	y	y	y	n	y	y	y
Local flap	0	0	0	0	0	0	1^1^
Free flap	0	0	2^2^	0	0	2^3^	0
Major amp	0	0	0	0	0	2^4^	1^5^
Minor amp	0	0	2^6^	0	0	0	0
Fasciotomy	1	1	1	0	1	1	1
Complications (e.g. flap loss)	1^7^	0	0	0	0	0	0

*ALT* anterolateral thigh, *amp* Amputation, *LD* Latissimus dorsi

^1^ Local LD flap

^2^ Free LD flap, free ALT flap for entry mark on head

^3^ Free LD flap for entry mark on head, free LD flap for right leg

^4^ Lower legs (1 Pirogoff)

^5^ Right arm

^6^ Forefoot right, 2 toes on left foot

^7^ Compartment

Apart from one patient suffering from compartment syndrome we did not see any other surgical complication such as flap loss.

## Implementation of an awareness project

Due to the increasing number of train surfers with correspondingly severe injuries, we found it essential to inform the relevant authorities and jointly launch an awareness project. In association with the Governmental Railways Agency of Austria an ongoing multimodal prevention program informing about the risks of high voltage trauma in print and online media as well as television channels was launched, especially targeting young people with the aim to increase awareness about the dangers of train surfing and climbing. Although the number of electrical injuries (EI) is low, these events are extremely traumatic, not only for the patients but also for the entire families. Therefore, the implementation of a self-support group at our clinic was initiated. This project was started by connecting three male patients (14, 17 and 19 years old), and their families. The patients and their relatives appreciated the opportunity to interact, share and process the traumatic experience.

## Discussion

Comparing normal burn patients to high voltage burn injuries from train surfers there are major differences: 3rd degree burn injuries marking the entry and exit point are typically seen, furthermore muscle damage leading to rhabdomyolysis, kidney failure is common as well as amputations or complex defect coverage especially of the entry and exit marks [13]. Patients suffer from cardiac arrest and arrhythmia. Secondary trauma, after falling from a train is common, leading to traumatic brain injury, fractures, spinal injuries and paraplegia.

Lumenta et al. [12, 16] noted that determining the best timing for surgery and managing soft tissue defects can be particularly challenging in high voltage injury patients compared to typical burn patients. This difficulty stems from the fact that high voltage injury patients often have additional health issues to consider.

In addition to the primary injuries, our patients have also experienced what we term “secondary trauma,” such as intracranial bleeding, fractures, and spinal injuries. These secondary traumas have occasionally been given priority for treatment by neurosurgeons or trauma surgeons, further complicating the overall management of these complex cases.

However, the exact approach to surgery will depend on the individual case and the specific needs of the patient, and in general, outcomes can vary widely depending on the severity and extent of the injury.

When looking at the study by Lumenta et al. [16] it can be seen that within a time frame from January 1994 to December 2008, 37 high-voltage accidents were treated, with only 12 being train surfers. This corresponds to roughly less than one patient per year. Within the last 2 years we observed an increasing number of patients, that were treated in our Center for Severe Burn Injuries due to train surfing or climbing. Compared to the data of Lumenta et al. [12, 16] our train surfing patients were generally younger. The extremities were more severely affected, and the length of stay in the intensive care unit was 24.8 days. The current flow in our patients, except for one, was vertical, similar to Lumenta et al.’s data. Lumenta et al. stated that another interesting point was the lower requirement for flap coverage and higher incidence of associated injuries; they hypothesized that due to the constant movement and speed of trains, the resulting risk for developing associated trauma was higher, while the contact time to an electric source was significantly shorter with less electric trauma in cases of survival. We cannot confirm this as most of our patients were not actually surfing on the train but climbing on it. Thus, the speed of the train did not significantly impact the injury patterns of our patients.

In their study Fordyce et al. [17] examined the work-related injuries from thermal, electrical, and chemical burns among electric utility workers. This population likely includes individuals engaged in various tasks within the utility industry. They indicated that burns accounted for a significant proportion of work-related injuries among electric utility workers, with nearly 13% of all medical claim costs attributed to thermal and electric shock injuries and highlighted the significant impact of burn injuries in occupational settings, emphasizing the importance of prevention strategies and occupational safety measures. In our study we identified specific high-risk behavior, such as train climbing and train surfing among adolescents, leading to high-voltage electrical injuries. Fordyce et al. [17] highlighted the significant impact of burn injuries in occupational settings, emphasizing the importance of prevention strategies and occupational safety measures. In their study Hussmann et al. [4] underline the mortality rate and severity of these injuries, which often result in extensive burn damage and long hospital stays. We focused on the severity of injuries, with a mean TBSA of 41.42% and a mortality rate of 14.29% among the study population. The mortality rate further emphasizes the life-threatening nature of these injuries and the critical need for effective management strategies.

The population in our study were patients suffering from train surfing burn and electrical injuries therefore we found only young male patients with an average age of 17.7 years. The study of Handschin et al. [18] also primarily included young males, consistent with the demographics typically associated with train surfing and climbing injuries. They aimed to provide a higher level of evidence regarding the surgical management of high-tension electrical injuries compared to thermal burns. Handschin et al.’s study emphasized the importance of proper defect coverage and decompression to mitigate complications associated with high-voltage injuries, which is in accordance with our study where surgical procedures including fasciotomy, amputation, and defect coverage using split skin grafts and flaps were used for defect coverage.

Electrical burn injuries mostly occur in an urban setting, Lin et al.’s study [19] provides a comprehensive analysis of subway-related fatalities in an urban setting, offering insights into the complexities of determining the manner of death in forensic investigations. By examining factors such as toxicology findings and circumstantial evidence, the study contributes to enhancing investigative practices and improving safety measures within subway systems. They conclude that a comparative analysis of injury characteristics, severity, and costs can provide information on the development of targeted interventions and safety protocols to reduce the incidence of burn injuries among electric utility workers and train surfers.

Kippe et al. [20] conducted a study to assess how the coronavirus disease 2019 (COVID-19) pandemic has affected individuals with mental health conditions. They reviewed the records of psychiatric emergency departments, focusing on patients with and without suicidal tendencies. The study revealed a rise in suicidal tendencies during the initial phase of the pandemic. During the second phase, this trend was notable among patients dealing with substance use disorders and bipolar disorder. Therefore, these individuals may face heightened suicide risks during the pandemic and require enhanced support and monitoring [20]. Our findings with increased number of train surfing patients could be a coincidence; however, psychosocial aspects of COVID-19 pandemic and lock down as well as isolation measures should also be considered.

Patients surviving the impact of electrical injuries face numerous burdens. With the increased survival of patients with large burns comes a new focus not only on medical but also on the psychological challenges and recovery that such patients must face. High voltage injuries can have significant psychological effects on individuals, such as depression, anxiety, or posttraumatic stress disorder, both in the short term and long term. In addition to these psychological effects, high voltage injuries can also increase the risk of substance abuse and suicidal ideation, as well as suicide attempts. Most burn centers employ social workers, vocational counsellors, and psychologists as part of the multidisciplinary burn team [21].

Interestingly, the literature provides only few data on adequate prevention treatment and outcome of these serious traumatic injuries and following complications [1, 7, 13]. Therefore, our study additionally discussed the implementation of an awareness project in collaboration with governmental agencies to educate the public about the dangers of train surfing and climbing. This included multimedia campaigns and the establishment of a support group for affected individuals and their families. The implementation of an awareness reflects a proactive approach to injury prevention, acknowledging the role of education and outreach in reducing the incidence of high voltage injuries among young individuals. By raising awareness about the dangers of train surfing and climbing, this initiative seeks to empower individuals to make safer choices and avoid unnecessary risks. Patients seemed to be unaware of the inflicted risks when mounting on the roof of a train. Unfortunately, none of our train climbing patients had been educated about the danger of electrical arcs.

Further research could explore the effectiveness of safety training programs, personal protective equipment, and engineering controls in mitigating occupational burn risks within the electric utility industry.

## Conclusion

The current study clearly shows that train surfing and climbing can result in severe injuries and fatal long-term consequences. Interestingly, all patients were young males. Adolescents seem to be curious about trains and tests of courage but do have a lack of knowledge about high voltage electrical currents as well as the hidden danger of light arcs. As a consequence, a pilot project was launched to raise awareness of the dangers of light arcs and the risk involved. Limitations of this study include the retrospective design, the relatively small number of patients as well as the short follow-up period.

Further research is needed to have a better understanding of risk factors associated with train surfing and train climbing as well as the effectiveness of prevention programs.
